# Transforming access to care for serious mental disorders in slums (the TRANSFORM Project): rationale, design and protocol

**DOI:** 10.1192/bjo.2022.584

**Published:** 2022-10-13

**Authors:** Swaran P. Singh, Sagar Jilka, Jibril Abdulmalik, Georgios Bouliotis, Rakesh Chadda, Olayinka Egbokhare, Rumana Huque, Gillian Lewando Hundt, Srividya Iyer, Obafemi Jegede, Neeru Khera, Richard Lilford, Jason Madan, Akinyinka Omigbodun, Olayinka Omigbodun, Tasneem Raja, Ursula M. Read, Bulbul Ashraf Siddiqi, Mamta Sood, Tanjir Rashid Soron, Helal Uddin Ahmed

**Affiliations:** Warwick Medical School, University of Warwick, Coventry, UK; and Coventry and Warwickshire NHS Partnership Trust, Coventry, UK; Warwick Medical School, University of Warwick, Coventry, UK; Centre for Child & Adolescent Mental Health & Department of Psychiatry, College of Medicine, University of Ibadan, Ibadan, Nigeria; Department of Psychiatry, All India Institute of Medical Sciences, New Delhi, Delhi, India; Department of Communication and Language Arts, University of Ibadan, Ibadan, Nigeria; Department of Economics, University of Dhaka, Dhaka, Bangladesh; Douglas Mental Health University Institute, Verdun, Quebec, Canada; Institute of African Studies, University of Ibadan, Ibadan, Nigeria; The Creative Gypsy, New Delhi, India; Institute of Applied Health Research, University of Birmingham, Birmingham, UK; Department of Obstetrics and Gynaecology, College of Medicine, University of Ibadan, Ibadan, Nigeria; Department of Political Science and Sociology, North South University, Dhaka, Bangladesh; Telepsychiatry Research and Innovation Network Ltd, Dhaka, Bangladesh; Adolescent and family Psychiatry Department National Institute of Mental Health, Dhaka, Bangladesh; on behalf of the **TRANSFORM** consortium*

**Keywords:** Serious mental illness, collaboration, faith and traditional healers, low-and-middle-income countries

## Abstract

This paper introduces the TRANSFORM project, which aims to improve access to mental health services for people with serious and enduring mental disorders (SMDs – psychotic disorders and severe mood disorders, often with co-occurring substance misuse) living in urban slums in Dhaka (Bangladesh) and Ibadan (Nigeria). People living in slum communities have high rates of SMDs, limited access to mental health services and conditions of chronic hardship. Help is commonly sought from faith-based and traditional healers, but people with SMDs require medical treatment, support and follow-up. This multicentre, international mental health mixed-methods research project will (a) conduct community-based ethnographic assessment using participatory methods to explore community understandings of SMDs and help-seeking; (b) explore the role of traditional and faith-based healing for SMDs, from the perspectives of people with SMDs, caregivers, community members, healers, community health workers (CHWs) and health professionals; (c) co-design, with CHWs and healers, training packages for screening, early detection and referral to mental health services; and (d) implement and evaluate the training packages for clinical and cost-effectiveness in improving access to treatment for those with SMDs. TRANSFORM will develop and test a sustainable intervention that can be integrated into existing clinical care and inform priorities for healthcare providers and policy makers.

Twelve per cent of the global disease burden is due to mental and behavioural disorders^1^ and more than 70% of this is experienced in low- and middle-income countries (LMICs).^2^ In Nigeria 12.1% of people have a lifetime rate of at least one DSM-IV disorder^3^ and in Bangladesh 16.1% of adults are estimated to suffer from some form of mental health problem.^4^ The mental health treatment gap, i.e. the difference between the number of people who need care and those who receive it, is reported at 80% for Nigeria, implying that only two out of every ten Nigerians with serious and enduring mental disorders (SMDs – defined as schizophrenia and other psychotic disorders, and severe mood disorders, often with comorbid substance misuse) are able to access care;^5^ in Bangladesh it is 92.3%, suggesting that fewer than one in ten who need mental healthcare are able to access it.^6^ In most LMICs public mental health systems do not receive adequate investment. For instance, in Bangladesh 2.64% of gross domestic product (GDP) is spent on health, which is the lowest in the South Asia region,^7^ and of the overall annual health budget, only 0.05% is designated for mental health.^8^ Yet the world's population living in slums reached 1 billion in 2018, and is projected to hit 3 billion by 2030, with the majority located in LMICs where the treatment gap is already huge.^9^

Help-seeking for SMDs in LMICs is pluralistic, with traditional and faith healers often being the initial, and sometimes the only, port of call (e.g.^10^). The preferred first contact for mental disorders is traditional and faith-based healers for 69% of adults in Nigeria^11^ and 30% in Bangladesh.^12^ Traditional or faith-based healing may alleviate symptoms in mood and anxiety disorders^13^ and provide valued social and spiritual support, but very little evidence exists that such practices improve care or outcomes for SMDs.^13–15^ On the contrary, reliance on traditional or faith-based systems can lead to treatment delays and poorer outcomes for people with psychotic illnesses.^16^ In many cases and across LMICs, it can also lead to coercion, including physical restraint, and human rights abuses.^17^

Within its Mental Health Gap Action Programme (mhGAP) the World Health Organization (WHO) has developed an intervention guide^18^ that aims to reduce the treatment gap by training non-specialist practitioners to screen for mental disorders, including serious mental illness (SMI), and provide treatment and appropriate referrals where needed. mhGAP strongly recommends the local contextualisation of training,^19^ especially cultural understandings of mental illness, which are a major determinant of help-seeking,^20^ but a systematic review of mhGAP in LMICs found that such contextualisation remains a challenge.^21^ Attempts at local adaptation of mhGAP have mainly been small scale.^22,23^ Large, community-based, stakeholder-driven, ‘bottom-up’ approaches to developing and testing interventions that are community-owned and community-delivered are lacking across LMICs.

Increasing people's access to psychiatric services without first determining local sociocultural understandings of mental illness, its causes and local help-seeking is unlikely to produce meaningful and sustainable change in the outcomes of SMI in LMICs. We need a deeper understanding of contextually embedded explanatory models and social determinants of mental illness and how these affect pathways to care, before settling on a solution such as ‘co-location’ of biomedical care at sites where patients seek traditional/faith-based healing.^24^ There is limited evidence on delivery and effectiveness of such collaborations, despite previous attempts to promote collaborations between faith-based healers and mental health services.^25,26^

## Aims and objectives of TRANSFORM

The TRANSFORM project aims to improve outcomes of persons with SMDs through better access to effective biomedical care by developing an innovative collaborative care model between traditional/faith healers, mental health professionals, primary care practitioners and community health workers (CHWs). We will develop and test community-delivered identification and referral pathways that recognise and engage with the experiences of mental ill health, existing beliefs about causation and treatment, and pluralistic help-seeking of slum-dwellers in Dhaka (Bangladesh) and Ibadan (Nigeria).

One of the key outputs of our project will be an intervention co-developed with key community stakeholders, which will improve the care of people with SMDs while protecting their human rights and meaningfully engaging with traditional and faith-based healers. Establishing such collaborations is not without particular and context-specific challenges, including long-held beliefs about mental health and illness and the power differentials between traditional healers and biomedical practitioners.^27^ Our innovative, bottom-up approach, whereby intervention and training are co-developed by healers, primary care workers and mental health practitioners in a spirit of mutual respect, seeks to address these challenges and promote participatory inclusiveness from all stakeholders.

## Method

### Design

**Fig. 1:**
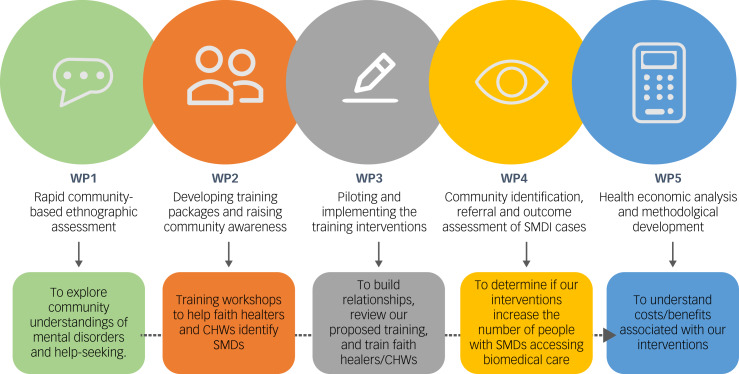
An overview of the TRANSFORM work packages outlining each package's goals. WP, work package; CHW, community health worker; SMD, serious mental disorder (psychotic disorders and severe mood disorders, often with co-occurring substance misuse).

The TRANSFORM study's design, depicted in Fig. 1, comprises five interlinked but distinct work packages (WPs), with data collection beginning in the first quarter of 2022 and a proposed study completion date of August 2024. The study will be conducted in accordance with ethical principles that comply with the Declaration of Helsinki and the International Council on Harmonisation's Harmonised Tripartite Guideline for Good Clinical Practice (GCP). All participants will provide informed consent before entering the study.

### Population

Our clinical population of interest are individuals (aged 18+) with SMDs living in deprived, urban neighbourhoods (slums) in Dhaka and Ibadan. Participants will be identified by healers and CHWs in WP4. We are not including individuals with a primary substance use disorder who do not have any SMD, as our pilot work has shown that people with substance use disorder do not seek traditional/faith healing and our intervention is therefore less likely to reach this specific population.

### Study sites

TRANSFORM brings together leading institutions across the UK, Asia, Africa and North America: the University of Warwick, UK; the National Institute of Mental Health and the Telepsychiatry Research and Innovation Network, Dhaka, Bangladesh; the University of Ibadan, Nigeria; and McGill University, Montreal, Canada.

We have chosen two sites, both in impoverished city areas, one in Dhaka, Bangladesh, and the other in Ibadan, Nigeria. These sites have been chosen because they both have well-established, well-patronised traditional healing services and well-developed mental health services within reach. At both sites, services offered by the CHWs and other primary and mental health services (both secondary and tertiary care) are typical of the traditional and faith-based healing pathways that operate in many LMICs. As a result, they provide an opportunity to test an integrated model and examine the barriers that arise from the very different ways in which traditional/local and universal/biomedical perspectives see the world. It cannot be assumed that integration will be unproblematic. Potential problems range from different epistemologies and taxonomies to perceived threats to livelihoods (e.g. competition for ‘customers’). There will also be social issues relating to gender, religion, identity, professional pride and autonomy, all of which may influence help-seeking for SMDs. Our project sites and our methodology offer an opportunity to understand these complex matters and provide sustainable solutions that have the potential to affect the understanding of, and attitudes towards, help-seeking for SMDs.

#### Ibadan, Nigeria

In Ibadan, the study will be conducted in the five urban local government areas (LGAs), which house adjacent and overlapping slum communities and have large numbers of traditional and faith healers who offer healing to people with SMDs. People predominantly of Yoruba ethnicity live in these five LGAs, which have a combined population of 1 338 659 inhabitants.^28^ Many practise religious syncretism through the combination of traditional African religions with Islam or Christianity. Several ancient and popular shrines, which serve as places for the treatment of mental illness, are found in the study area, alongside Islamic and Christian healing sites. Healing methods include divination, prayers and the use of herbal medicines. Apart from shrines, there are residential homes where people with mental illness stay to receive traditional healing. Many people with SMDs are homeless and roam around, especially in areas with reported high levels of psychoactive substance use and violence.

Nigeria operates a federal system with three tiers of government: a central administration, 36 states with their capital cities and 774 LGAs. In line with the three tiers of government, there are three tiers of healthcare delivery services: tertiary healthcare, provided principally by the federal government and consisting of specialised services in teaching and other specialist hospitals; secondary healthcare, provided in general hospitals run primarily by state governments; and primary healthcare, constitutionally the responsibility of local governments.

Basic healthcare services are provided in government-owned primary health clinics. There are between four and seven primary healthcare clinics in an LGA (district). The health workers who run the primary healthcare clinics are community health officers (CHOs) and community health extension workers (CHEWs), who are supposed to spend 70% of their time out of the primary healthcare centres and in the community. More infrequently, qualified public health nurses or midwives are also found in primary healthcare clinics. There are no dedicated mental health professionals at the primary healthcare level. Services are part of the public healthcare system and are ostensibly available free of cost. CHOs and CHEWs are permitted by law to identify, treat and prescribe medication for SMDs within the context of primary care using a standing order process. Owing to inadequate training and a lack of supervision, CHOs and CHEWs lack the capacity to manage persons with SMDs in primary healthcare and referrals are made to the nearest secondary or tertiary mental health centre.

The University College Hospital (UCH), Ibadan, a tertiary health centre (where the TRANSFORM investigators for Ibadan are based) is the flagship teaching hospital in Nigeria, with an array of clinical departments and specialist services, including psychiatry. This hospital is situated in the centre of an urban LGA close to the heart of the ancient city of Ibadan and is easily accessible by public transport. The Department of Psychiatry has 12 consultant psychiatrists, 30 resident doctors and a large multidisciplinary team consisting of psychiatric nurses, psychologists, social workers, occupational therapists and disability specialists. The hospital has 64 dedicated beds for adult psychiatry and an 8-bed adolescent in-patient unit. For persons without health insurance, healthcare is accessed by out-of-pocket payment.

The State Specialist Hospital, Ring Road Ibadan, at the secondary level of healthcare, is the second referral facility that is publicly run and provides specialist mental health services to the community. The government offers highly subsidised healthcare, so this hospital serves those who cannot afford care at the UCH. The facility has 20 beds for psychiatry, 3 psychiatrists, 2 medical officers, psychiatric nurses and social workers in the mental health unit.

#### Dhaka, Bangladesh

In Dhaka, the study will be conducted in the Korail Basti (*basti* meaning slum). Korail is one of the largest slums in Bangladesh, located in the centre of the city, encroaching on parts Dhaka's most affluent neighbourhoods. The area of the slum is claimed to be between 180 and 220 acres.^29^ The majority of Korail's population (estimated to be around 200 000)^30^ subsist below the poverty line and work in low-income jobs. They have limited access to the city's healthcare services and there is little connection with the surrounding major roads.^31^

There is widespread stigma related to mental illness in Bangladesh.^32^ Referrals of patients with SMDs to mental health specialists by primary healthcare providers are near non-existent, as are mental healthcare facilities.^33^ Many people living in the slums use traditional and faith-based healers. There are nine mosques within the Korail Basti, each with imams (Islamic priests) who are the ‘go-to people’ for mental health problems and support. One key religious healing practice is *Kalami* (a healing practice based on verses of the Qur'an). The healing aims to cure problems through communication with *jinn* (spirits), and treatments can also include giving patients *pani-pora* (blessed water) to exorcise the *jinn*.^34^

The National Institute of Mental Health (NIMH) is Bangladesh's leading public institution dedicated to the treatment of mental health problems, along with mental health policy formation, research and training of mental health professionals. The facility is located 7.3 km from the Korail Basti. It serves as one of the cheapest clinical options for the Korail community and employs over 300 personnel to deliver out-patient, in-patient and emergency medical services for child and adult mental disorders. As the largest psychiatric teaching hospital in the country, NIMH provides training in psychiatry, psychology and medicine as well as engaging in research activities and mental health policy formation.

NIMH has a total of 200 in-patient beds; 70% of these are non-fee-paying government-funded beds and the rest are private fee-paying beds. In-patient and out-patient admissions range across all units, including adult psychiatry and community and social psychiatry. In 2019, 58 846 patients were provided with service through the out-patient department.

Bangladesh has a pluralistic healthcare system where healthcare services are delivered by different public and private sectors, and we are also exploring ways to embed other referral systems in Dhaka as part of the study.

## Study delivery: proposed plan

### Work package 1: community-based ethnographic assessment (months 1–12)

In WP1, we will conduct a community-based ethnographic assessment using qualitative and participatory methods to explore local understanding of SMDs and help-seeking. We will use a variety of methods, including participatory mapping, free-listing and ranking exercises, walk-along interviews, observation and individual and natural group interviews to explore understandings and experiences of mental illness, poverty, daily life and the role of traditional and faith-based healing for SMDs, from the perspectives of people with lived experience of mental illness, caregivers, community members, healers, CHWs and health workers. Our findings will be used to co-design, with people with lived experience, caregivers, CHWs and traditional healing practitioners, training packages for screening, early detection and referral to mental healthcare for individuals (aged 18 and over) with SMDs. This will be used to develop a matrix of help-seeking pathways and barriers and enablers to mental healthcare.

### Work package 2: developing training packages for traditional/faith healers and community health workers; raising community awareness (months 12–18)

We will hold workshops with traditional/faith healers and CHWs to co-produce two training packages. The training package for healers will aim to help them identify signs of SMD in individuals who seek their care. We will identify best pathways for them to refer people with signs of SMD to the local primary care centre or to a CHW. The intervention will be designed not to replace faith and traditional healing practices with biomedical care, but rather to serve as a complement to their role.

For CHWs, we will modify the mhGAP intervention and guide^18^ based on local contextual factors identified through WP1 and based on previous work,^35^ including local taxonomies of mental illness and ideas on causation and help-seeking. The CHW training will upskill them to deliver community-based interventions, including screening and early identification of those with SMDs, psychoeducation, promoting medication adherence, referral and navigation to secondary care services, support for carers and community health promotion. It will also include a component pertaining to effectively liaising with traditional/faith healers, particularly for identifying those with SMDs who need additional care.

We will also co-produce media in various formats, such as posters and radio adverts, to engage communities with the intervention. The content and methods used will be informed by findings from WP1.

### Work package 3: piloting and implementing the training intervention (months 18–24)

We will introduce the training intervention and discuss how it can be used in day-to-day practice with CHWs, traditional healers, faith healers and primary care practitioners. In particular, traditional and faith healers will be trained as key informants who can offer people with SMDs and their caregivers the additional option of seeing a CHW. This will be in three phases:

#### Phase I: relationship-building

During this phase, we will discuss:

- recognition of the pivotal role of healers within the community and the value of their contributions
- the challenges of recognising SMDs and providing high-quality, rights-based care
- the importance of collaborating and working harmoniously for all stakeholders, particularly for people with SMDs and their caregivers.

#### Phase II: review of proposed training

This will cover:

- an overview of the proposed plan and training content
- willingness or reluctance of traditional and faith healers and CHWs to embark on training and ways to overcome any challenges
- iterative development of training packages based on feedback
- dealing with stigma of SMD, misperceptions about biomedical care and healers, financial constraints in accessing care, managing non-adherence to treatment or drop out from care, supporting households through psychoeducation and dealing with crises
- helping patients and families navigate the referral pathway
- use of community resources to facilitate recovery, such as self-care, supported employment, activities of daily living.

#### Phase III: delivery of training

Findings from phases I and II above, in addition to content generated from WP2, will allow the further refinement of the training package; the referral pathway and the support for ongoing care; and the identification of trainers. Following the refinement of the training package, two half-day interactive training workshops will be delivered to traditional practitioners and CHWs, with a half-day supervision session every 3 months. All training materials and delivery will be in the local languages (Bengali in Dhaka, and Yoruba in Ibadan).

### Work package 4: community identification, referral and outcome assessment of people with SMDs (months 24–48)

Our aim for WP4 is to determine whether our interventions lead to an increase in numbers of people with SMDs accessing biomedical care. We will further ascertain these individuals’ initial and follow-up mental health status and quality of life, reduction in caregiver burden, and changes in community attitudes and help-seeking for SMDs. We will collect data pre-intervention to identify the number of people with SMDs attending mental health services from the study site and their basic clinical and sociodemographic characteristics. Data collection systems will be in place for baseline and follow-up assessments of successive patients identified with signs of SMD and enrolled into the study over 12 months.

### Work package 5: health economic analysis and methodological development (months 24–48)

The overall research question for this WP5 is ‘What are the costs and benefits associated with investing in initiatives that facilitate relationships between traditional healers and the formal healthcare systems to improve access to care for those with SMDs?’ We will conduct primary data collection and analysis specific to the intervention developed in WP3. Our analyses will allow us to (a) generalise our work to interventions that aim to improve access more broadly, which will increase their impact and value to decision-makers; and (b) inform the development of mental health economics in LMICs and help address the need for capacity-building in this field.

### Capacity-building activities

We will identify capacity needs and co-develop and deliver high-quality capacity-building activities in Bangladesh and Nigeria, ranging from modules on research methodology to doctoral-level training and courses aimed at healthcare and leadership.

#### Doctoral research theses

The TRANSFORM programme is supporting three PhD candidates at the University of Warwick – one from Bangladesh and two from Nigeria – who will carry out their primary research activities in their home countries. These students, who were selected after a competitive process, commenced their doctoral studies in April 2022. The PhD topics include projects that are nested within TRANSFORM and stand-alone projects that are closely aligned to the TRANSFORM research priorities. At least two supervisors at the University of Warwick and one supervisor in the partner country are allocated to each student for their PhDs.

### Analysis plan

#### Qualitative analysis

For WP1, the collected transcribed data (both images and text) will be subjected to thematic analysis using qualitative software (NVivo 20). Analysis will be conducted first independently and then collaboratively to ensure reliability and validity within each site. The analysis will identify understandings and experiences of mental illness, poverty and daily life in the selected communities, as well as help-seeking for mental illness and practices of healers and health workers. The findings will inform the planning of the training interventions for CHWs and traditional/faith healers so that the interventions are contextually relevant and include the views of people with lived experience of mental illness and their families, healers and health professionals.

Findings from WP1 will also inform the qualitative evaluation of process and outcome after the intervention using the Qualitative Impact Protocol (QuIP).^36^ The QuIP is a method for qualitative impact evaluation for community-led and community-delivered interventions. It focuses on credible impact evaluation in relation to seeking to attribute outcomes to a specific intervention using interpretive methods. The QuIP uses a contribution analysis approach, combining quantitative monitoring of key indicators with qualitative, self-reported attribution of impact to provide sufficient evidence to test the theory of change behind the activity being evaluated.^37^ Importantly, although the QuIP evaluation can be designed by the study team, independent researchers, who are not associated with the intervention, will undertake the interviews to avoid confirmation bias and strengthen replicability.

The data collection for the process evaluation will include a replication of elements from the WP1 ethnographic assessment, including interviews with key participants on the referral pathway and group and individual interviews using participatory techniques. These will include people who accessed biomedical care and those who did not. In addition, interviews will be conducted with some of the previously interviewed natural groups when the listing and ranking and help-seeking matrix are repeated to assess whether there have been any changes in understanding. Data will be subjected to thematic analyses and these findings will inform the contextual interpretation of our quantitative findings.

#### Quantitative evaluation

In WP4, we will adopt a two-fold approach. Primarily, we will assess the impact of the proposed intervention on the (mental health) care pathway of individuals with SMDs and its potential effect on their health status. We will assess impact on the referral pathway by comparing referral rates made to the participating healers 12 months before and after intervention (training) is delivered. We will undertake a pre–post regression analysis to quantify the referral difference at the healer level after adjusting for healers’ and patients’ characteristics. Both the crude and the adjusted rate change and its corresponding 95% confidence interval will be provided.^38^ The change in referral rate will be the primary outcome of interest for this study.

We acknowledge that some participants may be directed to biomedical services by a healer but, for various reasons, may not actually access biomedical care. To further evaluate the impact of a healer's referral, we will try our best to trace their referrals and find out whether potential participants used mental health clinics.

In addition to the primary outcome, a number of patient-related secondary outcomes will be analysed. At this (patient) level, analysis will focus on clinical outcomes, functioning, participant and family satisfaction, medication adherence and attendance for clinical follow-up (Table 1 outlines the measures and time points for assessments).Uptake of healthcare will be evaluated longitudinally: at baseline (i.e. intake) and at months 1, 2 and 3. Similarly, clinical outcomes will be assessed longitudinally using the Global Assessment of Symptoms (GAS), Global Assessment of Functioning (GAF) and clinician-rated primary and secondary diagnoses. Carer burden will be assessed at baseline and post-intervention at month 3 using the Perceived Family Burden Scale (PFBS), and family satisfaction will be assess longitudinally using the Verona Service Satisfaction Scale (adapted to LMIC contexts). We will investigate service utilisation and associated costs using the abbreviated version of the Client Services Receipt Inventory (CSRI) – modified as used in our previous study.^39^ We will employ a linear mixed model to estimate longitudinal changes, adjusting for patient characteristics, time and baseline values/scores. Estimates and effect sizes will be reported, together with their 95% confidence intervals.

**Table 1:** Primary and secondary outcomes to be collected and their corresponding time points

		Assessment time point							
Domain	Tool	Pre-intervention	Baseline/intake	Month 1	Month 2	Month 3	Post-intervention	Analysis plan	Estimates reported
Number of individuals from study sites accessing biomedical care	Review of health record systems at NIMH in Dhaka (Bangladesh) and Ibadan (Nigeria)	✓					✓	Pre–post analysis	Referral rate and 95% CI
Number of referrals made by CHWs and traditional and faith healers	For each referral, CHWs will record date of referral and referral source (e.g. self, family member, faith or traditional healer)						✓	Pre–post analysis	Referral rate and 95% CI
Pathways to care (number and type of help-seeking contacts made prior to accessing biomedical care, and duration of untreated illness)	Nottingham Onset Schedule (NOS)		✓				✓	Pre–post analysis	Referral rate and 95% CI
Clinical characteristics of individuals accessing biomedical care	See assessment below	✓					✓	Longitudinal	Mean change since baseline
Sociodemographic profile									
	Age	✓	✓				✓	Longitudinal	Mean change since baseline
	Gender	✓	✓				✓	longitudinal	Mean change since baseline
	Education level		✓					Baseline only	descriptive only
	Religious background	✓	✓				✓	Longitudinal	Mean change since baseline
	Employment status		✓					Baseline only	Descriptive only
	Socioeconomic status		✓					Baseline only	descriptive only
	Family and marital status		✓					Baseline only	Descriptive only
	Area of residence	✓					✓	Longitudinal	Mean change
	Number of children <18 years old	✓	✓					Pre-intervention/baseline only	Descriptive only
Symptom severity	Global Assessment of Symptoms (GAS)		✓	✓	✓	✓		Longitudinal (baseline, months 1, 2 and 3)	Mean change (repeated measures)
Clinical/symptom profile	Clinician-rated primary and secondary diagnoses as recorded in charts		✓					Baseline only	Descriptive only
Level of functioning	Global Assessment of Functioning (GAF)		✓	✓	✓	✓		longitudinal (baseline, months 1, 2 and 3)	Mean change (repeated measures)
Physical health	Body mass index (BMI)		✓			✓		Longitudinal (baseline, month 3)	Mean change (repeated measures)
Carer burden	Perceived Family Burden Scale (PFBS)		✓			✓		Month 3 only	Descriptive only
Engagement with services	Record each month of whether patient contacted treatment provider and type of contact (phone, in-person, etc.).			✓	✓	✓		Longitudinal (months 1, 2 and 3)	Rate change (repeated measures)
Service utilisation and costs	Abbreviated version of modified version of Client Services Receipt Inventory (CSRI)		✓			✓		Longitudinal (baseline, month 3)	Mean/rate change (repeated measures)
Medication adherence	Medication Adherence Rating Scale (MARS)			✓	✓	✓		Longitudinal (months 1, 2 and 3)	mean change (repeated measures)
Patient and family satisfaction with services	Verona Service Satisfaction Scale (adapted to LMIC contexts)			✓	✓	✓		Longitudinal (months 1, 2 and 3)	Mean change (repeated measures)
Quality of life	EuroQoL five dimensions questionnaire (EQ-5D)		✓			✓		Longitudinal (baseline, month 3)	Mean change (repeated measures)

CHW, community health worker; NIMH, National Institute of Mental Health; LMIC, low- and middle-income country.

We will follow checklists such as the STROBE Statement to report our observational research. We are intending to develop a dedicated statistical analysis plan (SAP) with detailed description of the analyses undertaken (primary and sensitivity), the handling of missing values and model-assumption violations. The alpha = 0.05 significance level will be used in all analyses.

### Management

The University of Warwick is the sponsor organisation. A programme manager is responsible for the day-to-day management of the project and supports the principal investigator on the project's overall delivery. The programme manager, principal investigator and work package leads are responsible for the overall management of the study and meet monthly by video conference chaired by the principal investigator. The project steering committee (PSC), comprising the programme manager, principal investigator, co-investigators and work package leads, provides overall strategic direction, ensures good governance, provides scientific input, guides capacity-building activities and provides financial probity when required.

An independent external scientific advisory board (ESAB) of international experts will monitor and assess the study conduct and progress. Both the PSC and ESAB will meet annually with the consortium to assess the scientific quality of the research and provide feedback.

### Ethics

Informed consent will be obtained from all participants as appropriate, before their enrolment into the study. We will develop a clinical care pathway for participants that will ensure safety, protection of confidentiality and privacy. The research team will preserve the confidentiality and anonymity of participants taking part in the study and fulfil transparency requirements under the UK General Data Protection Regulation for health and care research, with equivalent guidance from Nigeria and Bangladesh. The study may be subject to inspection and audit by the University of Warwick under their remit as sponsor and by other regulatory bodies to make sure of adherence to good clinical practice (GCP) and the UK Policy Framework for Health and Social Care Research.

The TRANSFORM team will apply for ethics approval specific to each work package from the University of Warwick's Biomedical and Scientific Research Ethics Committee, Coventry, UK, and the research ethics committees of all participating organisations. The ethical conduct of the study is monitored throughout by the University of Warwick and each participating organisation.

### Dissemination

We will engage with our strong network of stakeholders involved in mental health services, such as clinicians, academics, service providers, policy makers, governmental and non-governmental agencies, community groups, patient and carer advocacy groups, media agencies and the creative arts industry, to support dissemination activities and public engagement across a variety of academic and non-academic audiences. We will (a) develop and maintain an up-to-date project website and social media page(s) (to target different audiences/stakeholders); (b) present study findings at national and international psychiatric and mental health scientific conferences and in peer-reviewed publications; and (c) disseminate our findings as broadly as possible in Bengali and Yoruba through film, radio and street theatre developed with local creative partners and civil society advocacy groups. We will work closely with our partners to identify the dissemination and public engagement approaches that will work best in these settings, to increase the impact of our research, to further engage the target communities, raise awareness, reduce stigma and provide information on plural healthcare for SMD.

### Patient and public involvement

We have strong and ongoing engagement with various internal and external stakeholders involved in mental healthcare. Patient and public involvement (PPI) is at the heart of TRANSFORM, and the entire study was conceived with people with lived experience of SMDs, their families, faith healers, charity organisations and the wider community. WP1 uses participatory approaches to understand local and contextual understandings of mental health, illness and help-seeking behaviour from the perspective of patients and carer groups and voluntary/charity/third-sector organisations, ensuring representation of hard-to-reach groups. The community were involved in proposing the research objectives and in the overall study design and will be central to the implementation of our intervention; and co-author O.J. is an expert on Yoruba history and *Ifa* divination, a traditional healer and co-investigator. All researchers and partners in TRANSFORM also have an extensive record of patient and carer involvement in research. We have a robust programme for monitoring and governance – research, ethics and financial – and our programme will adhere to the highest levels of ethical practice and probity, including PPI.

## Discussion

The TRANSFORM study plans to build on lessons learned from previous research in this area, particularly from the University of Ibadan, which is a centre of excellence and WHO Collaborating Centre for mental health research. The University of Ibadan has been the site of a significant number of influential studies on SMI and help-seeking which have led to several high-quality publications, including, but not limited to:

- INTREPID 1 and 2 – social epidemiological and qualitative research on methods of community case finding and help-seeking pathways for psychosis (funded by the Medical Research Council) (e.g.^40^)
- PAM-D – formative qualitative research on local concepts of mental health and explanatory models (funded by the US National Institute of Health) (e.g.^27^)
- COSIMPO – a cluster randomised controlled trial of a collaborative care model for psychosis with traditional and faith-based healers and primary care mental health workers (funded by the US National Institute of Mental Health).^25^

TRANSFORM will build on these studies, elaborating and extending understanding through its distinctive methodology and approach, including:

- evaluating impact and effectiveness as the primary outcome
- targeting people with all SMDs, including severe depression, not just psychosis
- developing a help-seeking pathway and provision from the community, through various care pathways (primary and secondary care) with follow-up
- developing a clear referral pathway to existing mental health services
- training primary care workers/CHWs to screen for SMDs and refer, rather than using specialist mental health nurses, in line with the integration of mental healthcare into primary care
- increasing collaboration, understanding and respect between mental health workers, including psychiatrists, nurses and CHWs, and healers of different types through a strong focus on participatory methods and community engagement
- comparison between Nigeria and Bangladesh rather than another country within Africa.

TRANSFORM also aims to innovate methodologically through:

- using mixed methods, in particular ethnography and participatory methods, in addition to qualitative interviews and quantitative methods
- involving the community, including people with lived experience, caregivers and other stakeholders, from the outset and throughout the process through community advisory groups and other participatory methods, with a view to building in sustainability
- using results of initial ethnographic research on existing help-seeking practices as well as the activities of healers and primary health workers to inform training (WP2), intervention (WP3) and evaluation (WP4 and WP5).
- co-producing training with CHWs, mental health workers and healers
- conducting both process and outcome evaluation using mixed methods, including ethnography and qualitative interviews to understand how, why and for whom the intervention was or was not successful.

## Conclusions

The sizeable differences in social and cultural context, lived experience, sociodemographics, infrastructure and availability of resources mean that models of care developed in high-income countries cannot be simply translocated to LMICs.^39^ TRANSFORM will co-develop a novel and sustainable referral pathway for people with SMDs to access biomedical care, while engaging with the benefits of existing pluralistic help-seeking pathways. We will tailor evidence-informed interventions to the local sociocultural context to address the increasing burden of SMDs in Nigeria and Bangladesh. By developing a novel intervention that is regionally relevant, TRANSFORM will develop and test sustainable interventions that can be integrated into existing clinical care and generate data (including health economic data) that will inform priorities for healthcare providers and policy makers.

## Data Availability

Data availability is not applicable to this article as no new data were created or analysed in this report.
